# Bioprospecting *Chromobacterium violaceum* for bioremediation: an alternative to environmental lead pollution

**DOI:** 10.1007/s11274-026-05190-8

**Published:** 2026-09-04

**Authors:** Feliphe Lacerda Souza de Alencar, Julio Alejandro Navoni, Daniel Chaves de Lima, Rodrigo Soares Caldeira Brant, Viviane Souza do Amaral

**Affiliations:** 1 Post graduation Program in Development and Environment, Federal University of Rio Grande do Norte, Natal, RN Brazil; 2 Parasitology and Microbiology Department, Federal University of Rio Grande do Norte- UFRN, Natal, RN Brazil; 3 Federal Institute of Education, Science and Technology - IFRN, Ipanguaçu, RN Brazil; 4 Mass Spectrometry Facility RPTH02, Carlos Chagas Institute, Fiocruz, Curitiba, Paraná Brazil; 5 Department of Cellular Biology and Genetics, Federal University of Rio Grande do Norte, Natal, RN Brazil

**Keywords:** *Chromobacterium violaceum*, Bioremediation, Pollution, Lead

## Abstract

Lead pollution is a major environmental concern, but current decontamination technologies remain limited due to high costs. Therefore, alternative biotechnological processes have been successfully developed and applied due to their reduced cost and lower aggressiveness in the environment. The remarkable adaptive versatility of *Chromobacterium violaceum* in metal-contaminated environments makes this bacterium a promising candidate for Pb bioremediation. Therefore, the reference strain *C. violaceum* ATCC 12,472 and the environmental isolate *C. violaceum* SCV1, the first strain of this species isolated from a Brazilian area with natural Pb occurrence, were evaluated for Pb resistance under different Pb concentrations and exposure times. Pb biosorption was assessed by scanning electron microscopy, while strain-specific protein profiles were characterized using tandem mass spectrometry-based proteomic analysis. The results obtained revealed the potential of *C. violaceum* to perform lead bioremediation. Scanning electron microscopy analysis confirmed the biosorption of lead by *C. violaceum* strains. *C. violaceum* SCV1 was able to remove up to 40% more lead concentration when compared to ATCC 12,472 which suggested adaptation through natural selection process of *C. violaceum* SCV1. Proteome analysis revealed 1531 proteins, of which several are candidates for lead bioremediation. This is the first study on the resistance proteomics of *C. violaceum* against lead. The acclimatization of the bacteria linked to the identification of several proteins related to: biosorption; efflux and ionic uptake (bioaccumulation); biomolecule transport; and biomethylation, point out to this organism as a potential lead bioremediation agent.

**Supplementary Information:**

The online version contains supplementary material available at https://doi.org/10.1007/s11274-026-05190-8.

## Highlights

*C. violaceum* SCV1 strain shows higher Pb resistance and removal than ATCC 12,472;

Up to 54% Pb removal achieved by *C. violaceum* under in vitro conditions;

Proteomics reveals metabolic reprogramming under Pb exposure;

Pb detox involves biosorption, efflux, transport and biomethylation;

Novel proteins identified as candidates for Pb bioremediation.

**Supplementary Information:**

The online version contains supplementary material available at https://doi.org/10.1007/s11274-026-05190-8.

## Introduction

The impacts of pollution on aquatic ecosystems are an important problem faced by contemporary societies due to the production and environmental fate of toxic substances (Dantas et al. 2013; Afzal et al. 2014; Hassaan et al. 2016; Azevedo et al. 2018; Rodell et al. 2018). Metal contamination remains a persistent environmental challenge because of its non-biodegradable nature, long-term persistence, potential for bioaccumulation, and detrimental effects on microbial communities and ecosystem functioning (Briffa et al. 2020). Lead (Pb) stands out among the metals identified as risk agents, as it is a non-essential, non-degradable toxic metal, and easily absorbed in different organisms. Accumulating evidence indicates that Pb exposure induces oxidative stress, disrupts enzymatic pathways, alters cellular signaling, promotes apoptosis, and contributes to teratogenic, mutagenic, and carcinogenic effects, as well as genomic instability. These adverse outcomes reinforce the need for efficient strategies to remove Pb from contaminated environments. (Jaishankar et al. 2014; Kushwaha et al. 2018).

Some organisms such as bacteria species have shown adaptation and/or resistance mechanisms against different substances such as metals.

These mechanisms are increasingly investigated through integrated omics approaches, particularly proteomics, which enables the identification of molecular responses associated with metal tolerance, transport, stress adaptation, and bioremediation potential (Lacerda et al. 2021; Zhang et al. 2022). Microbial resistance to metals involves multiple coordinated mechanisms, including efflux pumps, complexation, biosorption, bioaccumulation, enzymatic detoxification, extracellular polymeric substance production and intracellular sequestration. Growing evidence indicates that these mechanisms can be investigated through integrated omics approaches, particularly proteomics, enabling the identification of novel microbial candidates for sustainable bioremediation (Ahemad and Malik 2012; Dixit et al. 2015; Li et al. 2015; Kothari et al. 2017). *Chromobacterium violaceum* is among the microorganisms with biotechnological potential due to its metabolic and adaptive versatility involved in resistance to a variety of metals such as arsenic, iron, cadmium, zinc, manganese, aluminum, mercury and gold (Alencar et al. 2019; Lima et al. 2021). However, the molecular mechanisms underlying adaptation to Pb exposure remain poorly understood. No study has systematically compared the physiological and proteomic responses of an environmental isolate naturally adapted to lead-rich conditions with those of the reference strain ATCC 12,472. Moreover, an integrated analysis combining comparative proteomics, lead biosorption assays, ultrastructural characterization, and protein–protein interaction network analysis has not yet been performed to identify molecular candidates associated with Pb tolerance and bioremediation in *C. violaceum*. (Alencar et al., 2016 and 2017; Najiah et al. 2008; Tran et al. 2011).

Therefore, a better understanding regarding the respective resistance profiles, bio-sorption and complexation mechanisms of *C. violaceum* is important in the context of the bioprospecting as a tool for lead bioremediation, as well as to identify and characterize proteins of biotechnological interest. However, the integration of comparative proteomics and protein–protein interaction network analysis to investigate lead adaptation in environmental isolates of *C. violaceum* remains largely unexplored (Armengaud 2022; Zhou et al. 2022). Thus, this study compared two *Chromobacterium violaceum* strains: the reference type strain ATCC 12,472, originally isolated in Malaysia (Hungria et al. 2004), and SCV1, a novel environmental strain isolated by the authors from a natural lead-occurring area in the semiarid region of northeastern Brazil, specifically in the municipality of Lajes Pintadas, in the state of Rio Grande do Norte, an area geologically characterized by the occurrence of uranium-rich pegmatites which naturally results in radon, a radioactive gas, and finally become to lead^210^ as its last decays element and environmental stable form (Barillet et al. 2011; Campos et al. 2013; Dantas et al. 2013). Since *C. violaceum* SCV1 is described here for the first time, its physiological and proteomic characteristics under lead exposure remain unknown. Therefore, the comparative analysis performed in this study aimed to identify potential adaptive traits associated with its environmental origin and evaluate its potential for lead bioremediation. To comprehensively evaluate the Pb bioremediation potential of the two *Chromobacterium violaceum* strains, complementary phenotypic and molecular approaches were employed. Lead resistance was evaluated as an indicator of the ability of each strain to survive under metal stress, biosorption assays were used to determine their capacity to remove Pb from the surrounding environment, and comparative proteomic analysis was performed to identify the molecular mechanisms associated with Pb adaptation and tolerance.

## Materials and methods

### Isolation and identification of the SCV1 strain

The *C. violaceum* SCV1 strain was isolated from water samples collected at the Riacho da Cachoeira reservoir (6°08′30″ S, 36°06′54″ W), located in the municipality of Lajes Pintadas, Rio Grande do Norte State, northeastern Brazil. Water samples were collected from five sampling points along the reservoir in October 2014, during the dry season. The region has a semi-arid climate characterized by low annual precipitation, high temperatures, and marked seasonal variation in water availability. The reservoir was selected because of the natural occurrence of Pb associated with uranium-rich pegmatitic formations in the Borborema Province (Campos et al. 2013; Dantas et al., 2014).

All samples were collected under aseptic conditions and transported under refrigeration to the laboratory for processing. The reference ATCC 12,472 strain was supplied by the Molecular Biology and Genomics Laboratory of the Federal University of Rio Grande do Norte. Bacterial isolation and microbiological identification were performed according to the procedures described in the *Standard Methods for the Examination of Water and Wastewater*. Briefly, water samples were initially filtered through a membrane filter. The membrane was then transferred to Tryptic Soy Broth (TSB) and incubated at 35 ± 2 °C for 24 h. Following bacterial growth, an aliquot of the culture was streaked onto nutrient agar plates and incubated again at 35 ± 2 °C for an additional 24 h to obtain isolated colonies.

All bacterial isolates were identified using the automated VITEK^®^ 2 GN system (bioMérieux, France). Briefly, well-isolated colonies were suspended in sterile saline solution to a turbidity equivalent to 0.5 McFarland standard. The bacterial suspensions were subsequently inoculated into VITEK^®^ 2 GN identification cards specific for Gram-negative bacilli, and species identification was performed automatically according to the manufacturer’s instructions. The isolate also exhibited the characteristic violet pigmentation of *Chromobacterium violaceum*. Additionally, the species identification was corroborated by the subsequent proteomic analysis, in which LC-MS/MS data were matched against the *Chromobacterium violaceum* protein database, resulting in the identification of numerous proteins assigned to this species.

### Lead resistance assessment

The *C. violaceum* ATCC 12,472 and *C. violaceum* SCV 1 strains were used to obtain the bacterial inoculum.

The isolation and identification of *C. violaceum* SCV 1 strain was performed following the methodology recommended by the Standard Methods for Examinations of Water and Wastewater (Alencar et al. 2019). The experiments to determine the minimum inhibitory concentration (MIC) and evaluate Pb bioremediation capacity were performed in Erlenmeyer flasks containing Luria–Bertani (LB) broth inoculated with previously cultured *C. violaceum* ATCC 12,472 and SCV1 strains at a 1:100 inoculation ratio, resulting in a final volume of 100 mL, following an adaptation of the methodologies described by Lima et al. (2014) and Alencar et al. (2019). Prior to inoculation, bacterial suspensions of *C. violaceum* ATCC 12,472 and SCV1 were prepared from fresh cultures and standardized by viable cell counting. The initial bacterial density was adjusted to approximately 10⁶ colony-forming units per milliliter (CFU mL⁻¹). Cultures were incubated under the experimental conditions described below, and bacterial growth was monitored throughout exposure to increasing concentrations of Pb(NO₃)₂.

The culture medium was supplemented with sterile, autoclaved lead nitrate [Pb(NO₃)₂] to obtain final concentrations of 0.5, 1, 2, 3, 4, 5, 6, and 7 mM. These concentrations were selected considering the environmental relevance of Pb contamination and the legal threshold established by CONAMA Resolution No. 430/2011. Exposure times of 0, 7, 12, 24, and 30 h were defined according to the growth stages of *C. violaceum*, and bacterial growth was monitored using the microdrop method (Herigstad et al. 2001; Alencar et al. 2019). All experiments were performed in biological triplicate. Bacterial growth was evaluated using a generalized linear mixed model (GLMM), with optical density (OD) as the response variable. Strain, lead concentration, incubation time, and concentration-by-time interaction were included as fixed effects. Three independent biological replicates were performed for each strain, and each replicate was monitored throughout the experiment across all incubation times and lead concentrations. Replicate identity was included as a random intercept to account for between-replicate variability while accommodating the correlation among repeated observations obtained from the same biological replicate. The model was fitted assuming a Gamma distribution for the response variable with an identity link function, which adequately described the distributional characteristics of the data while preserving the original measurement scale. Model parameters were estimated by maximum likelihood using the Laplace approximation, and statistical significance was assessed using Type III Wald χ² tests with a significance level of α = 0.05. Model adequacy was evaluated by inspecting residual diagnostics and convergence before statistical inference.

The robustness of the statistical inference was further assessed through a retrospective simulation-based power analysis using the final fitted GLMM. Statistical power was estimated by parametric Monte Carlo simulation (1,000 iterations), reproducing the fitted model and evaluating the effects of strain, lead concentration, incubation time, and the concentration-by-time interaction. Power was defined as the proportion of simulated datasets in which each fixed effect remained statistically significant at α = 0.05.

All analyses were conducted within a Python 3 computational environment (Python Software Foundation, Wilmington, DE, USA). Statistical models were fitted in R version 4.2.2 (R Foundation for Statistical Computing, Vienna, Austria) through the *rpy2* interface using the *lme4* package for mixed-model estimation and the *car* package for Type III Wald χ² tests. The retrospective power analysis was implemented using custom R routines integrated into the same Python workflow, ensuring full computational reproducibility of the statistical analyses.

### Lead removal

A standard concentration of 3 mM was established according to the data obtained in the respective growth curves presented by *C. violaceum* ATCC 12,472 and SCV 1 strains against Pb(NO_3_)_2_ to compare the respective in vitro removal capabilities of the lead in both strains. The culture medium, bacteria, and metal set were evaluated after 30 h of inoculation (the end of the stationary phase of bacterial growth). All treatments were carried out in biological triplicate. The concentration of each metal in the digestion extract was subsequently determined in an atomic absorption spectrophotometer. The respective content of the metal in the bacterial biomass (mg L^-1^) was determined, as well as the metal removal rate from the culture, as adapted by Moreira et al. (1993) and Pot et al. (1994).

### Scanning electron microscopy

The bacterial suspension samples (*C. violaceum* ATCC 12472 and *C. violaceum* SCV 1) from the lead removal experiment mentioned above were analyzed by Scanning Electron Microscopy. Briefly, prior to the sample preparation protocol, 10 µL of poly-L-lysine (0.1%) was deposited and spread on glass coverslips so that the bacteria adhered to its surface. After drying the poly-L-lysine, 20 µL of bacterial suspension was added over the coverslips and dried again. Then the samples were fixed in Karnosvsky solution for 36 h, washed with cacodylate buffer (0.02 M), post-fixed with osmium tetroxide for one h, and finally dehydrated in an acetone gradient. Metallization with gold was performed to increase the electrical conductivity of the samples, following the protocol described by Vicentin et al. (2016).

### Protein extraction

The incubation times were selected according to the biological objective of each experiment. Proteomic and SEM-EDS analyses were performed after 12 h of incubation, corresponding to the end of the exponential growth phase, when metabolically active cells are expected to exhibit primary molecular and structural responses to lead exposure. In contrast, lead removal was quantified after 30 h of incubation, corresponding to the stationary phase, to evaluate the overall bioremediation efficiency after prolonged contact between bacterial cells and Pb.

*C. violaceum* ATCC 12,472 and SCV 1 strains were comparatively analyzed regarding exposure (test) and non-exposure to lead (negative control) to extract their respective total proteins for further proteomic analysis.

The test exposed the strains to 3 mM of Pb(NO_3_)_2_, ensured the colonies growth for 12 h (end of the exponential phase of bacterial growth). The strain samples were then centrifuged at 2880 RCF at 4 °C for 30 min. The supernatant was discarded, and all samples were resuspended in 50 mM EDTA pH 8.0. A new centrifugation was performed under the same conditions, and the supernatant was discarded again. The samples were subsequently washed in a 10 mM Tris-HCl solution, pH 8.5, centrifuged and discarded according to the previous procedure. The cells pellets were lysed in 400 µL of extraction buffer consisting of 7 M Urea, 1 M Thiourea, 50 mM DTT, 0.5% CHAPS and 30 mM Tris-HCl pH 8.5. Then, samples were washed twice with 1 ml of acetone to precipitate the proteins, followed by mechanical agitation and centrifuging at 10,000 RCF at 4 °C for 5 min. Finally, the total proteins were solubilized in the same extraction buffer in a volume of 300 µL, according to the methodology described by Lima et al. (2014).

### Protein quantification, SDS-PAGE and band excision

Total protein extracts from *C. violaceum* ATCC 12,472 and SCV1 were quantified using the Bradford assay (Bradford, 1976). For proteomic analysis, 50 µg of total protein from each sample was mixed with SDS-PAGE sample buffer, denatured under appropriate conditions, and loaded onto a 13% polyacrylamide resolving gel. Electrophoresis was performed for 30 min at 50 mA to separate proteins according to their molecular mass. Following electrophoresis, no individual protein bands were selected based on differential staining intensity or electrophoretic profile. Instead, the entire protein lane corresponding to each biological replicate was excised into gel slices for proteomic analysis.

### Digestion in gel and peptide extraction

The resulting gel slices were subjected to in-gel tryptic digestion, and the extracted peptides were subsequently analyzed by LC-MS/MS. The lanes excised from the respective samples of *C. violaceum* strains were initially decolorized in microcentrifuge tubes with a solution containing 25 mmol L^-1^ ammonium bicarbonate in 50% ethanol. The tubes were then shaken in a thermomixer at 25 °C (72 RCF) until the solution was saturated, at which point the supernatant was removed and discarded. The procedure was repeated until the gel was completely discolored. The samples were dried in a speed-vac for 7 min (vacuum pressure = 0.1). Next, 12.5 ng µL^-1^ trypsin solution diluted with 50 mmol L^-1^ ABC solution was added to the gel at the time of use. The gel remained with trypsin for 20 min at 4 °C. The excess of trypsin solution was removed and discarded. Finally, enough digestion buffer was added to cover the gel, and the sample was again incubated at 37 °C for 16 to 18 h, as per protocol ICC-POP.116 provided by the Carlos Chagas Institute, Fiocruz/PR.

### Peptide extraction

The liquid contained in the tubes after digestion in gel with the interval of 16 to 18 h was poured into a new 2 mL eppendorf tube (step - A). Then, an extraction solution (Water, 3% trifluoroacetic acid [TFA] and 30% acetonitrile) was added in a sufficient amount to cover the gel to extract the peptides from the samples, but without exceeding 400 µL. The samples were stirred in a thermomixer for 10 min at 25 °C (72 RCF) and the supernatant was collected and transferred to the 2 mL eppendorf tube (step - B) from step A, and then this step was repeated once more, totaling two repetitions. Acetonitrile was subsequently added to the samples until the gel was covered and a new stirring step was performed in a thermomixer for 10 min at 25 °C (72 RCF). The supernatant was transferred to the same tube of step B, with this procedure repeated one more time totaling two repetitions. A spin was then performed to recover all liquid from the samples. Finally, the samples were dried (supernatant) in a speed-vac (vacuum pressure = 0.1) at 45 °C up to 10 to 20% of the original volume in order to remove acetonitrile and proceed to purify the peptides as per protocol ICC -POP.116 provided by the Carlos Chagas Institute, Fiocruz/PR.

### Purification of peptides in Stagetip - Stop and Go Extraction in Tip

The peptides from protein digestion in gel were purified/desalted. The StageTip-C18 was initially prepared with a C18 membrane disk (3 M EmporeTM) being placed on a clean Petri dish. The C18 membrane was drilled, and a small portion was extracted, which was then transferred to a tip (2-200 µL). The samples were diluted in AD solution (0.1% formic acid, 5% dimethyl sulfoxide and 5% acetonitrile) according to the total mass to be injected for further analysis in a mass spectrometer (LC-MS/MS) as per protocol ICC-POP.119 provided by the Carlos Chagas Institute, Fiocruz/PR.

### Mass spectrometry analysis

Following in-gel digestion and peptide purification, the samples were analyzed using an Easy-nLC 1000 nano-liquid chromatography system coupled to an LTQ Orbitrap XL ETD mass spectrometer (Thermo Scientific) to characterize the proteomes of *C. violaceum* ATCC and SCV 1 strains under both lead-exposed and non-exposed conditions. Proteomic analyses were performed in collaboration with the Mass Spectrometry Platform of the Carlos Chagas Institute, Oswaldo Cruz Foundation (FIOCRUZ), Paraná, Brazil.

Chromatographic separation was carried out on an Easy-nLC 1000 system using solvent A (0.1% formic acid and 5% DMSO in water) and solvent B (0.1% formic acid and 5% DMSO in acetonitrile) at a flow rate of 250 nL min⁻¹. Peptides were separated using a linear gradient from 5% to 40% solvent B over 240 min on a 15-cm analytical column (75 μm internal diameter) packed with 3-µm C18 particles.

The LTQ Orbitrap XL ETD hybrid mass spectrometer was operated under the following conditions: (i) full MS (MS1) scans were acquired in the Orbitrap analyzer over an *m/z* range of 300–2,000 at a resolution of 60,000, using a lock mass of *m/z* 401.922718; (ii) MS/MS (MS2) spectra were acquired in the linear ion trap using a data-dependent acquisition method with the ten most intense precursor ions selected for fragmentation; (iii) dynamic exclusion was enabled with an exclusion duration of 90 s; (iv) instrument tune settings included nanospray ionization (NSI), capillary temperature of 175 °C, ion trap full AGC target of 30,000, and FTMS full AGC target of 1,000,000; and (v) analyses were performed in positive ion mode with a spray voltage of 2.70 kV, source current of 100 µA, capillary voltage of 35 V, and tube lens voltage of 100 V.

After LC-MS/MS acquisition, peptide and protein identification were performed using MaxQuant (version 1.6.3.4) with the Andromeda search engine. MS/MS spectra were searched against the *Chromobacterium violaceum* ATCC 12,472 reference proteome downloaded from UniProt (4,397 protein entries; database version dated January 16, 2020). Protein identification was accepted using a false discovery rate (FDR) of 1% at both peptide-spectrum match (PSM) and protein levels. Label-free quantification (LFQ) was subsequently analyzed in Perseus (version 1.6.3.4), and differential protein abundance between treatments was evaluated using Student’s *t*-test (*p* < 0.05) (Tyanova et al. 2016; Cox and Mann 2008).

### Analysis of hypothetical or uncharacterized proteins

To better understand the functions of uncharacterized proteins in *C. violaceum* ATCC 12,472 and SCV1 strains after the proteome analysis, local alignments of those considered hypothetical were carried out to re-record their functions or to identify functional domains. The evaluated parameters were based on the databases of non-redundant protein sequences (nr) using the blastp (protein-protein BLAST) algorithm through the website: https://www.ncbi.nlm.nih.gov.

### Systems biology analysis - protein-protein interaction networks

The protein-protein interaction analyses were performed using the Cytoscape software (3.7.1) (Shannon et al. 2003). The String-db app (www.string-db.org) (Szklarczyk et al. 2023) integrated with Cytoscape was initially used to create the network. A total of 187 UNIPROT codes referring to the proteins of the groups were assessed. The corresponding network model was constructed based in those proteins differentially expressed in every strain.

A color gradient was used to map the Fold-change of proteins in the network. The analysis of gene ontology was performed by the String-db plugin. The Biological Process category was enriched and added to the network nodes. The centrality, node degree and Betweenness indices were computed with the Centiscape plugin (Scardoni et al. 2014) integrated with the Cytoscape software. Bottleneck proteins are the ones with the highest node degree and betweenness values.

## Results

### Resistance profile

*C. violaceum* SCV1 was isolated and identified in the municipality of Lajes Pintadas for the purpose of bioprospecting *C. violaceum* for lead bioremediation for the first time. This regional strain had its lead resistance profile analyzed in vitro and compared to the ATCC 12,472 strain.

To understand the influence of the strain, the level of lead exposure and the exposure time on bacterial growth a generalized linear mixed model was performed using Gamma distribution and identity link function. Both the strain (X^2^_(1)_: 8.90; *p* < 0.001), time (X^2^_(1)_:447.5; *p* < 0.001), lead concentration (X^2^_(1)_: 596.7; *p* < 0.001) and the interaction between lead concentration and exposure time (X^2^_(1)_: 141.6; *p* < 0.001) significantly influenced the bacterial growth (represented by the measured optical density). Table 1 describes the estimated parameters of the model.

**Table 1:** Parameter estimates of the GLMM model considering as predictor variables the strain along with as covariates the lead concentration and exposure time on the bacteria growth

				95% Confidence Intervals			
Names	Effect	Estimate	SE	Lower	Upper	z	*p*
STRAIN	SCV1 - ATCC 12,472	0.07579	0.02540	0.02577	0.12581	2.98	0.003
Pb (mM)	CONC	-0.13111	0.00537	-0.14167	-0.12054	-24.43	< 0.001
TIME(h)	TIME	0.02481	0.00117	0.02250	0.02712	21.16	< 0.001
CONC ✻ TIME	CONC ✻ TIME	-0.00596	4.94e-4	-0.00693	-0.00498	-12.07	< 0.001

**Fig. 1:**
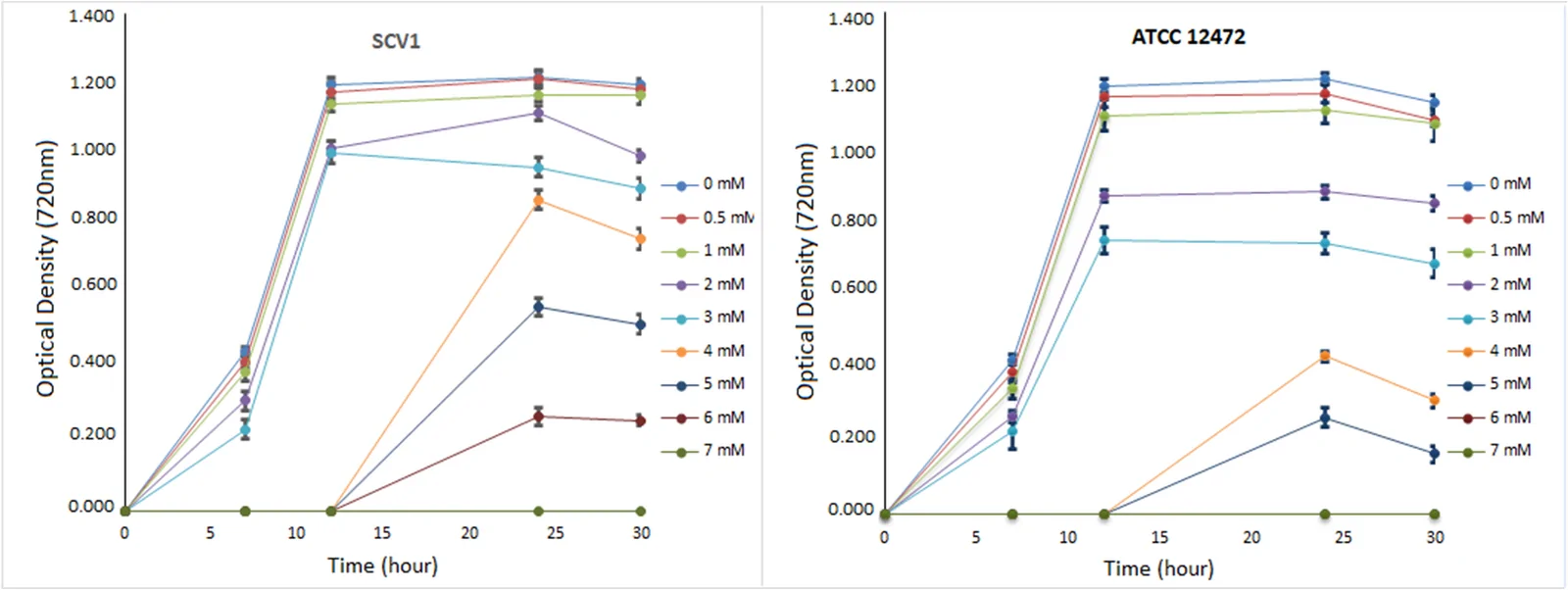
Resistance profile of the SCV1 and ATCC 12,472 *C. violaceum* strains when exposed to different lead concentrations and growth intervals

The strain influenced the growth response in the tested experimental conditions. On average, *C. violaceum* SCV1 grew 8% more than the ATCC reference strain. In addition, Lead concentrations reduce the growth in 13% per every additional unit of metal. Time influenced the bacterial growing fact that was negatively altered by the raising of metal concentration (Fig. 1). For instance, both strains showed similar patterns of resistance when exposed to the lowest lead concentrations (0.5 mM to 3 mM) between the intervals of 0, 7, 12, 24 and 30 h of growth. However, *C. violaceum* SCV 1showed a more efficient lead resistance pattern than *C. violaceum* ATCC 12,472, especially between concentrations from 4 to 5 mM. Comparing the influence of the time, bacterial growth was progressively delayed, indicating an extension of the lag phase in the first 7 h of growth from 0.5 to 3 mM concentrations, at 4 mM concentration only after 12 h of growth, and 6 mM during 24 h of growth. *C. violaceum* ATCC 12,472 was not able to survive at the 6 mM concentration, unlike *C. violaceum* SCV 1, which in turn did not resist the 7 mM lead concentration.

A retrospective simulation-based power analysis showed that the fitted model had high statistical power (Higher than 99%) to detect the effects of lead concentration, incubation time, and their interaction. In contrast, the overall strain effect showed moderate statistical power (72.0%; 95% CI: 69.1–74.8%), indicating lower sensitivity for detecting small differences between strains while providing high sensitivity to detect the main treatment effects investigated in this study.

### Assessment of bioremediation capacity

Based on the initial tests aimed at assessing the adaptive *C. violaceum* capacity against lead exposure, the bioremediation potential of both strains was assessed. Efficiency of lead removal was assessed after 30 h of exposure to 3 mM lead (a viable concentration for the growth of both strains). The data showed that the indigenous *C. violaceum* SCV1 was able to remove more lead than *C. violaceum* ATCC 12,472 strain (expressed as mean and 95%IC) 54.20% [44.6–63.8] vs. 32.66% [24.2–41.2] of the initial lead test concentration, respectively.

### Scanning electron microscopy and bacterial biosorption

In addition to assessing the ability to remove lead in vitro by *C. violaceum* strains, the capacity for lead complexing and biosorption was assessed, because they are relevant adaptive mechanisms for assessing the bioremediation potential of bacteria against lead. As a result, both strains showed similar behavior for lead biosorption in this analysis.

**Fig. 2:**
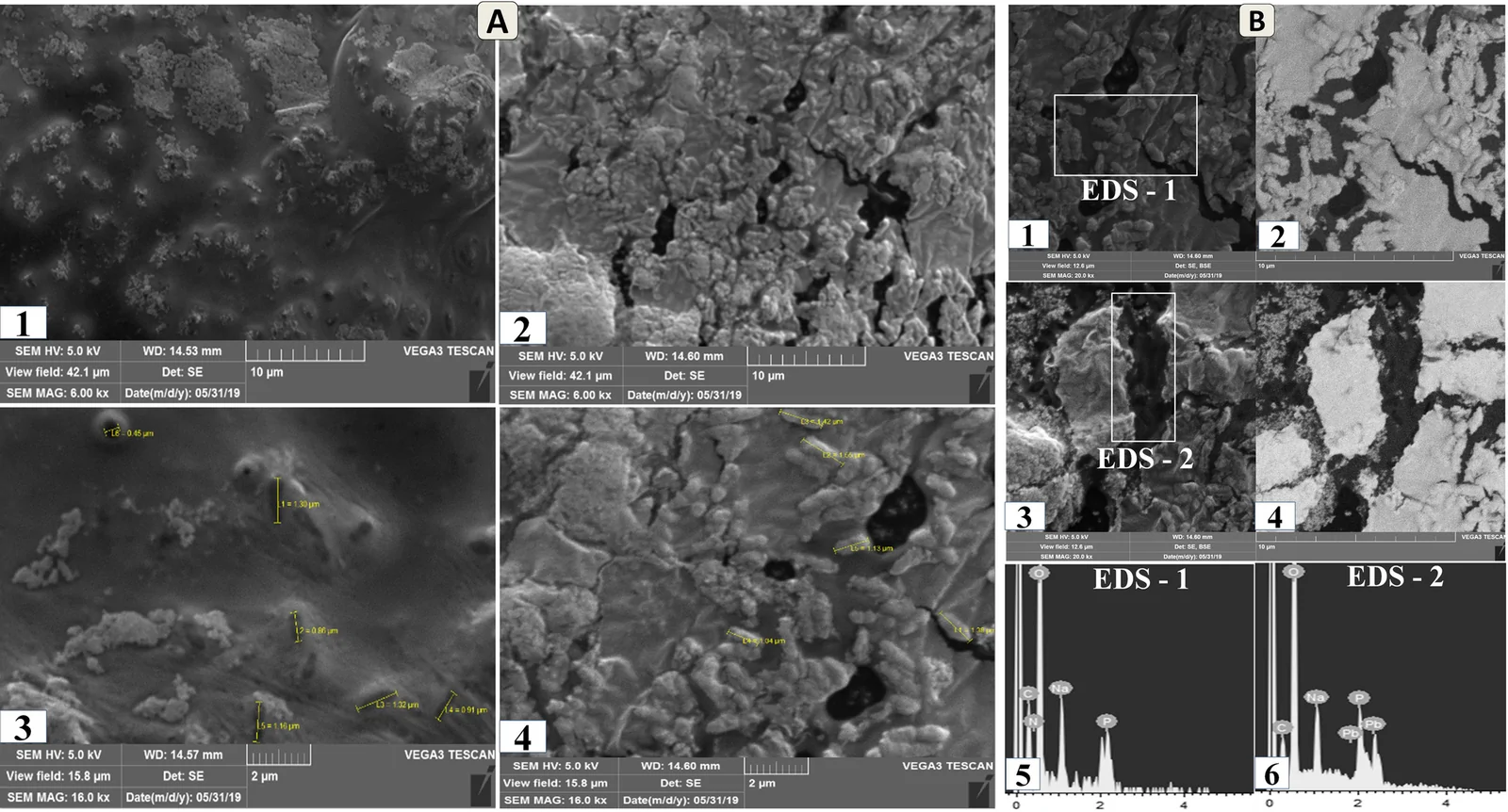
**A** Scanning electron microscopy - *C. violaceum* ATCC 12,472 grown in the presence and absence of lead. (1 and 3) - Bacterial sample exposed to lead (test), with rod-shaped and/or coconut-bacillus *C. violaceum* structures, with the darkened hue showing the lead adsorption; (2 and 4) - Bacterial sample not exposed to lead - negative control, in the images there are *C. violaceum* bacillary structures with a grayish-white hue, free from lead. The analysis shows the general behavior of *C. violaceum* for lead biosorption, however without distinguishing the respective strains tested, these showed similarities when performing Scanning electron microscopy. **B**. Analyzes of Backscattered Electrons and Dispersive Energy Spectroscopy - EDS in *C. violaceum* in the presence and absence of lead. (1) - bacterial sample not exposed to lead - negative control. In the images there are bacillary *C. violaceum* structures with a grayish-white hue, delimited - EDS - 1; (2) - Backscattered electron analysis, where the η emission demonstrates a direct relationship with the atomic number of the elements present in the sample. There is also a homogeneous contrast in the regions where the bacterial strain is located; (3) - micrograph showing the elementary mapping by EDS - 1 for the region delimited in figure (1), indicating the bacterial basic composition - Carbon, Nitrogen, Oxygen and Phosphorus. (4) - *C. violaceum* sample exposed to lead, in which it is possible to notice bacillary structures with a darkened hue, delimited - EDS - 2. (5) - Backscattered Electron analysis, giving the image a composition contrast. *C. violaceum* bacillary structures exposed to lead are located in the darkened regions. (6) - micrographs showing elementary mapping by EDS - 2 for the regions delimited in figure (4), indicating the presence of lead adsorbed to the bacteria

The bacteria presented rod or coco-bacillus shapes with dimensions between 0.4 and 0.9 μm in diameter from 1.5 to 3.0 μm in length. The images obtained revealed a large amount of *C. violaceum* structures in both conditions (presence of lead) (Fig. 2A). It is interesting to note that after 12 h of exposure (the end of the exponential phase of bacterial growth) to lead at 3 mM, the scanning electron microscope analysis showed that *C. violaceum* has an ability to withstand the exposure without undergoing significant changes on its structure and maintaining its bacillary shape. Additionally, the cellular structures identified in *C. violaceum* a semi-quantitative microanalysis of the chemical elements was performed and a chemical mapping of bacterial surfaces by Energy Dispersion Spectroscopy (EDS) was constructed. Thus, a Back-Scattering Electron (BSE) analyzes provided topographic and composition images (contrast in function of the atomic number of the elements present in the samples) in different analyzed regions in view of the presence of adsorbed metal in the bacterial strains (Fig. 2B).

### Proteomics analysis

**Fig. 3:**
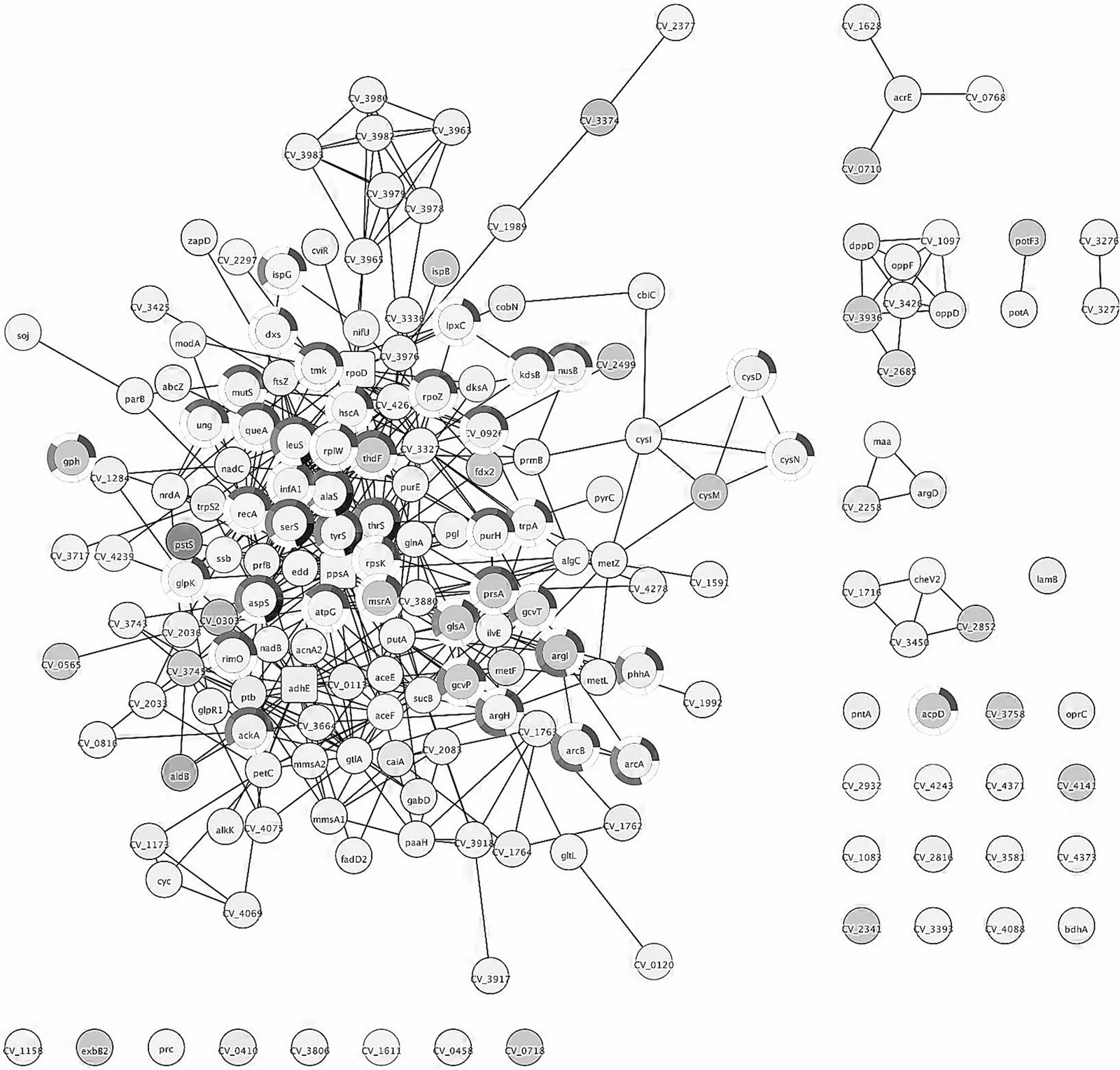
Venn diagram comparing the identified proteins in both strains and experimental conditions

A differential proteomic analysis was performed aiming to identify proteins related to lead bioremediation. Analysis of the proteome of *C. violaceum* ATCC 12,472 and SCV 1 strains revealed the bacteria complexity adaptation to lead 3 mM during 12 h of exposure. A total of 1531different proteins were identified with statistical significance in both strains represented through a Network Diagram (Fig. 3).

Additionally, evaluating the *C. violaceum* molecular behavior for the purpose of bioprospecting for lead bioremediation, it was found that the main molecular functions detected among the proteins identified in *C. violaceum* ATCC 12,472 and SCV 1 strains post-exposure to lead were related to signaling pathways, oxidative stress, cell cycle, cell and protein metabolism, gene expression, extracellular matrix organization, RNA metabolism, DNA replication and transport. Furthermore, 21.3% of proteins in *C. violaceum* SCV 1 had transport-related functions, mainly associated with the ABC-type transport system, bio-molecules transport, electron and ion transport, as well as transmembrane transport and 60% of the proteins identified had complexation and bio-molecular binding mechanisms, of which 28.8% were associated with metals such as iron, zinc, copper and manganese and magnesium. Cell transport systems were specified in about 12% of proteins in the ATCC 12,472 strain, and just as in *C. violaceum* SCV 1, they were mainly associated with the ABC type transport system for the bio-molecule transport, as well as functions related to efflux, ion channel formation and electron transport and 70% of the proteins identified in ATCC 12,472 had bio-molecular complexation and binding mechanisms, of which 35.5% were associated with metals such as iron, zinc, copper and manganese and magnesium (supplementary material).

The proteins identified in both *C. violaceum* strains were also evaluated for their respective functions to verify possible mechanisms, processes, and reactions that favor lead bioremediation. However, 23 uncharacterized proteins were identified in *C. violaceum* ATCC 12,472 after lead treatment, whereas 17 proteins were also identified with unknown functions in *C. violaceum* SCV 1 under the same treatment conditions. After local alignment (BLASTP) and consultation of the respective CDD domains (Conserved Domain Database), it was found that 39.1% of the 23 proteins evaluated in the ATCC 12,472 strain had Missing Conserved Domain (MCD) or Domain of Unknown Function (DUF), whereas in *C. violaceum* SCV 1 it was found that 52.9% of the 17 hypothetical or uncharacterized proteins presented either MCD or DUF, as shown in Table 2.

**Table 2:** Proteins identified in *C. violaceum* after lead exposure, for which the bioinformatics analysis (BLASTP and CDD) confirmed the absence of a conserved domain in its structure (Missing conserved domain - MCD) or domain with unknown function (Domain of Unknown Function - DUF)

ORF	UniprotKB	Genomic context^*^	Domain
ATCC 12,472			
CV_1311	Q7NYG3	N-acetylmuramoyl-L-alanine amidase (CV_1309); Class C beta-lactamase (CV_1310); Hypothetical proteins (CV_1312); DJ-1/PfpI family protein (CV_1313)	MCD
CV_2383	Q7NVG0	Hypothetical proteins (CV_2381); Aspartate/tyrosine/aromatic aminotransferase (CV_2382); TetR/AcrR family transcriptional regulator (CV_2384); Mechanosensitive ion channel (CV_2385)	MCD
CV_2658	Q7NUN9	Cytochrome P450 (CV_2656); Hypothetical proteins (CV_2657) AAA family ATPase (CV_2659); PaaI family thioesterase (CV_2660)	MCD
CV_2975	Q7NTS8	Sigma-70 family RNA polymerase sigma factor (CV_2973); DNA repair protein RadA (CV_2974); SDR family oxidoreductase (CV_2976); NAD(P)-binding protein (CV_2977)	MCD
CV_3039	Q7NTL5	FCD domain-containing protein (CV_3037); ADP-glyceromanno-heptose 6-epimerase (CV_3038); D-glycero-beta-D-manno-heptose-7-phosphate kinase (CV_3040); UDP-glucose/GDP-mannose dehydrogenase family protein (CV_3041)	DUF
CV_3099	Q7NTF7	Lipoyl(octanoyl) transferase LipB (CV_3096); Lipoyl synthase (CV_3097); Hypothetical protein (CV_3098); Magnesium-translocating P-type ATPase (CV_3100)	MCD
CV_4324	Q7NQ18	YbjQ family protein (CV_4322); Hypothetical protein (CV_4323) Murein tripeptide/oligopeptide ABC transporter ATP binding protein OppF (CV_4325); Oligopeptide ABC transporter ATP-binding protein OppD (CV_4326)	DUF
CV_3560	Q7NS66	Histone deacetylase family protein (CV_3558); Hypothetical protein (CV_3559); Cysteine synthase A (CV_3561); Ion transporter (CV_3562)	MCD
CV_2950	Q7NTV3	Hypothetical protein (CV_2948, CV_RS14435); GNAT family N-acetyltransferase (CV_2951, CV_2952)	DUF
**SCV 1**			
CV_3773	Q7NRK8	NCS2 family permease (CV_3771); Adenine phosphoribosyltransferase (CV_3772); Hypothetical protein (CV_3774); DUF1700 domain-containing protein (CV_3775); PadR family transcriptional regulator (CV_3776)	MCD
CV_2519	Q7NV27	Bifunctional tetrahydrofolate synthase/dihydrofolate synthase (CV_2518); Hypothetical protein (CV_RS12315) ATP-binding cassette domain-containing protein (CV_2520); Transporter substrate-binding domain-containing protein (CV_2521)	MCD
CV_0349	Q7P165	Phage virion (CV_0347);Lytic transglycosylase domain-containing protein (CV_0348); Tail sheath protein (CV_0350); DUF1834 family protein (CV_0351)	MCD
CV_0338	Q7P175	DMT family transporter (CV_0336); Phage tail protein (CV_0337); Hypothetical protein (CV_0339); Tail fiber assembly protein (CV_0340)	MCD
CV_0010	Q7P251	DUF1484 family protein (CV_0008, CV_0009); Hypothetical protein (CV_0011); DUF2235 domain-containing protein (CV_0012)	DUF
CV_0009	Q7P252	DUF1484 family protein (CV_0008, CV_0010); ATP-binding protein (CV_RS23225); Hypothetical protein (CV_0011)	DUF
CV_0008	Q7P253	DUF2357 domain-containing protein (CV_0007); ATP-binding protein (CV_RS23225); DUF1484 family protein (CV_0009, CV_0010)	DUF
CV_0397	Q7P117	Methyl-accepting chemotaxis protein (CV_0395); PAS domain S-box protein (CV_0396); Biopolymer transporter ExbD (CV_0398); MotA/TolQ/ExbB proton channel family protein (CV_0399)	DUF
CV_0063	Q7P1Z9	LysR family transcriptional regulator (CV_0061); DMT family transporter (CV_0062); Hypothetical protein (CV_0064); DEAD/DEAH box helicase (CV_0065)	DUF

* Describes the location of the gene on the chromosome in non-sequential coordinates, providing information about the gene and its neighbors, the exon organization of the gene and the RefSeqs used to represent RNA products and their coding regions

In addition, a total of 131 proteins were identified in *C. violaceum* ATCC 12,472 with significant changes in their abundance (up and down regulation) after lead exposure. Moreover, about 20% of the up-regulated proteins in *C. violaceum* ATCC 12,472 strain were considered hypothetical or have not yet been characterized. The regulated proteins present in *C. violaceum* ATCC 12,472 performed molecular functions related to metal complexation and ionic transport system such as the ABC system, efflux system and transmembrane transport, or mechanisms associated with genetic material repair, oxidative stress and cell resistance (Table 3).

**Table 3:** Proteins identified in the ATCC 12,472 strain whose relative quantity has undergone a statistically significant change in its abundance after lead exposure. The data generated from the analyzes in the Perseus program using the log2 of the LFQ intensity of at least 2 replicates of the test and two replicates of the negative control, applying the Student’s t-test with *p* < 0.05

Protein	UniProtKB	Gene	Fold Change**
Aldehyde dehydrogenase (NAD)	Q7P121	aldB	− 22.0
Probable indolepyruvate ferredoxin oxidoreductase, subunit alpha	Q7P1B0	CV_0303	− 20.0
Glycine dehydrogenase (decarboxylating)	Q7NSJ5	gcvP	− 13.1
Probable ABC transporter ATP-binding	Q7NUL2	CV_2685	− 10.7
Methylenetetrahydrofolate reductase	Q7NZF6	metF	− 9.0
Aminomethyltransferase	Q7NSJ3	gcvT	− 8.9
Acyl-CoA dehydrogenase	Q7NWA6	caiA	− 6.2
Probable short-chain dehydrogenase	Q7NVT4	CV_2258	− 5.7
Ornithine carbamoyltransferase	Q7NWJ6	argF	− 5.6
Probable ATP-binding component of ABC transporter	Q7NZ22	dppD	− 5.2
Probable cytochrome-c oxidase, subunit II	Q7NYU9	CV_1173	− 4.7
Maltoporin	Q7NSX1	lamB	− 4.6
Probable acyl-coA carboxylase subunit	Q7MBF6	CV_1762	− 4.4
Biopolymer transport	Q7M7G2	exbB1	− 4.2
Cobalamin biosynthesis	Q7NXQ5	cobN	− 4.2
Probable propionyl-CoA carboxylase	Q7NX65	CV_1764	− 3.9
Acriflavin resistance protein E	Q7NXJ9	acrE	− 3.6
Outer membrane protein OprC complement	Q7NZM7	oprC	− 3.4
Probable aspartate/glutamate racemace	Q7P1U9	CV_0113	− 3.4
Acetylornithine aminotransferase	Q7NVT6	argD	− 3.3
Sulfate adenylyltransferase subunit 2	Q7NVN6	cysD	− 3.3
Probable methyl-accepting chemotaxis	Q7NXB1	CV_1716	− 3.2
Efflux transporter outer membrane subunit	Q7NXJ8	CV_1628*	− 3.1
Probable PhoH-related	Q7NU87	CV_2816	− 3.1
Succinate-semialdehyde dehydrogenase [NAD(P)]	Q7NR57	gabD	− 3.1
OmpA-like domain-containing	Q7NR09	CV_3976	− 3.0
Probable hydrolase	Q7NYJ0	CV_1284	− 3.0
Cell division	P60012	zapD	− 2.9
ImpA_N domain-containing	Q7NR22	CV_3963	− 2.9
Probable cytochrome c5	Q7NQR8	CV_4069	− 2.9
Cell division protein FtsZ = 243,365	Q7NQ04	ftsZ	− 2.5
Oligopeptide transport system ATP-binding	Q7NQ16	oppD	− 2.5
Probable enoyl-CoA hydratase	Q7NWA7	CV_2083	− 2.5
O-succinylhomoserine sulfhydrylase	Q7NUH3	metZ	− 2.4
Methylmalonate-semialdehyde dehydrogenase	Q7NWA5	mmsA1	− 2.4
Nicotinate-nucleotide pyrophosphorylase	Q7NXN8	nadC	− 2.4
Acetate kinase	Q7NRN5	ackA	− 2.3
Glycerol-3-phosphate regulon repressor	Q7P1V0	glpR1	− 2.3
Oligopeptide ABC transport system ATP-binding	Q7NQ17	oppF	− 2.3
Probable L-carnitine dehydrogenase-like	Q7NR67	CV_3917	− 2.3
Probable enoyl-CoA hydratase	Q7NX66	CV_1763	− 2.3
Sulfate adenylyltransferase subunit 1	Q7NVN5	cysN	− 2.3
Type VI secretion system contractile sheath small subunit	Q7NR06	CV_3979*	− 2.3
Type VI secretion system membrane subunit TssM	Q7NR05	CV_3980*	− 2.3
Acyl-CoA synthetase	Q7NZM4	alkK	− 2.2
Cytochrome-c oxidase	Q7NPV6	cyc	− 2.2
HTH tetR-type domain-containing protein	Q7NSJ9	CV_3425	− 2.2
Probable chemotaxis protein CheA	Q7NSH6	CV_3450	− 2.2
Probable general secretion pathway protein L	Q7NRH5	CV_3806	− 2.2
Ubiquinol-cytochrome c reductase	Q7NQX9	petC	− 2.2
Isocitrate dehydrogenase [NADP]	Q7NRW4	CV_3664	− 2.1
3-hydroxybutyryl-CoA dehydrogenase	Q7NWA4	paaH	− 2.0
Precorrin-8X methylmutase	Q7NXQ9	cbiC	− 2.0
Type VI secretion system contractile sheath large subunit	Q7NR07	CV_3978*	− 2.0
Type VI secretion system lipoprotein TssJ	Q7NR03	CV_3982*	− 2.0
Bifunctional purine biosynthesis protein	Q7P0M1	purH	− 1.9
Branched-chain-amino-acid aminotransferase	Q7NW96	ilvE	− 1.9
N5-carboxyaminoimidazole ribonucleotide mutase	Q7P1Q2	purE	− 1.9
Probable ClpA/B-type chaperone	Q7NR20	CV_3965	− 1.9
Type VI secretion system baseplate subunit TssK	Q7NR02	CV_3983*	− 1.9
4HBT domain-containing	Q7P0V7	CV_0458	− 1.8
Glyco_trans_2-like domain-containing	Q7NRA3	CV_3880	− 1.8
Probable binding protein component of ABC dipeptide transporter	Q7NSJ8	CV_3426	− 1.8
Probable glutaryl-CoA dehydrogenase	Q7NR66	CV_3918	− 1.8
Probable transcriptional regulator	Q7NQP9	CV_4088	− 1.8
Phosphoenolpyruvate synthase	Q7NRS1	ppsA	− 1.8
Ribose-phosphate pyrophosphokinase	Q7NQS9	prs	− 1.8
Serine–tRNA ligase	Q7NY99	serS	− 1.8
Spermidine/putrescine import ATP-binding	Q7NQN5	potA	− 1.8
Argininosuccinate lyase	Q7P1U7	argH	− 1.7
Long chain fatty-acid CoA ligase	Q7NY16	fadD2	− 1.7
Nucleotide-binding	Q7NST4	CV_3336	− 1.7
Tyrosine–tRNA ligase	Q7NTI2	tyrS	− 1.7
1-deoxy-D-xylulose-5-phosphate synthase	Q7NUK5	dxs	− 1.6
Chromosome partitioning	Q7P0A3	parB	− 1.6
Glutamine synthetase	Q7NS38	glnA	− 1.6
Methylmalonate-semialdehyde dehydrogenase	Q7NY38	mmsA2	− 1.6
Pyruvate dehydrogenase E1 component	Q7P0P0	aceE	− 1.6
Sulfite reductase hemoprotein, beta subunit	Q7NS53	cysI	− 1.6
WHy domain-containing	Q7NZ39	CV_1083	− 1.6
50 S ribosomal protein L23	Q7NQF4	rplW	− 1.5
Aconitate hydratase	Q7NWD6	acnA2	− 1.5
Probable lysine decarboxylase	Q7NWJ7	CV_1992	− 1.5
Thymidylate kinase	Q7NRQ7	tmk	− 1.5
Citrate synthase	Q7NZ52	gtlA	− 1.4
tRNA modification GTPase MnmE	Q7NPT9	mnmE	− 1.4
L-aspartate oxidase	Q7NR54	nadB	− 1.3
Phosphoenolpyruvate-protein phosphotransferase	Q7NZV5	CV_0816	− 1.3
Threonine–tRNA ligase	Q7NYC6	thrS	− 1.3
Carboxy-terminal processing protease	Q7NSR6	prc	− 1.2
Polyphosphate: AMP phosphotransferase	Q7NQR2	CV_4075*	− 1.2
Probable NADH-ubiquinone oxidoreductase	Q7NWF7	CV_2033	− 1.2
Alanine–tRNA ligase	Q7NXM2	alaS	1.1
Aspartate–tRNA(Asp/Asn) ligase	Q7NRP0	aspS	1.2
S-adenosylmethionine: tRNA ribosyltransferase-isomerase	Q7NZD4	queA	1.2
Phosphogluconate dehydratase	Q7P1R9	edd	1.4
Probable glutathione transferase	Q7NPW9	CV_4373	1.5
Probable KpsF/GutQ family	Q7NSU3	CV_3327	1.5
Homoserine dehydrogenase	Q7NZC6	metL	1.5
Bifunctional protein PutA	Q7NXT8	putA	1.6
Probable cellobiose phosphorylase	Q7NYW4	CV_1158	1.6
CreA family	Q7NRR3	CV_3717*	1.7
Transcriptional regulator	Q7NVG6	CV_2377*	1.8
JmjC domain-containing	Q7NS46	CV_3581	1.8
YceI domain-containing	Q7NSZ2	CV_3277	1.8
ABC transporter ATP-binding	Q7NV26	abcZ	1.8
Ribosomal protein S12 methylthiotransferase	Q7NYA1	rimO	1.9
Rick_17kDa_Anti domain-containing	Q7NPX1	CV_4371	1.9
Probable haloacid dehalogenase-like hydrolase	Q7NXN5	CV_1591	1.9
YceI domain-containing	Q7NSZ3	CV_3276	2.0
Phosphoglycolate phosphatase	Q7NW10	CV_2180	2.1
Probable ribokinase	Q7NQ62	CV_4278	2.1
Transcription antitermination	Q7NVF2	nusB	2.2
Transcriptional activator, LuxR/UhpA - regulators	Q7NQP7	cviR	2.2
6-phosphogluconolactonase	Q7P1R7	pgl	2.2
3-hydroxybutyrate dehydrogenase	Q7P073	bdhA	2.4
Acetate kinase	Q7NXU5	ackA	2.5
Enoyl-[acyl-carrier-protein] reductase [NADH]	Q7NRN7	CV_3743	2.6
Peptide chain release factor 2	Q7NZ61	prfB	2.7
Phenylalanine-4-hydroxylase	P30967	phhA	2.8
Ornithine carbamoyltransferase, catabolic	Q7NRK0	arcB	2.8
Probable porin	Q7NWK0	CV_1989	3.1
Aldehyde-alcohol dehydrogenase	Q7NYY5	adhE	3.1
Arginine deiminase	Q7NRJ9	arcA	3.3
Tryptophanyl-tRNA synthetase	Q7NRR5	trpS2	3.5
NAD(P) transhydrogenase, alpha subunit	Q7P1W1	pntA	3.6
Probable peroxiredoxin/glutaredoxin family protein	Q7NWF4	CV_2036	3.7
Phosphate acetyltransferase	Q7NRN6	ptb	4.1
Probable bacteriophage tail core	Q7P104	CV_0410	5.5
Phasin_2 domain-containing	Q7NSP6	CV_3374	18.1
Phosphate-binding	Q7NZI4	pstS	32

* reannotated proteins after functional alignment. ** The data consists of converting the fold change log2 of the respective strains to absolute values, which indicate the number of times a given protein was regulated after lead exposure, with the values being positive referring to up-regulation and negative referring to down-regulation

Furthermore, a predominance of transferase, hydrolase and oxidoreductases enzymes was observed in both *C. violaceum* ATCC 12,472 and SCV 1 strains post-exposure to lead in evaluating proteins with catalytic activity were found (supplementary material).

A total of 43 proteins were identified in t *C. violaceum* SCV 1 with significant alteration in their abundance, of which approximately 5% were uncharacterized proteins. The proteins regulated in this strain generally showed molecular characteristics like the ATCC 12,472 strain, showing a metabolic reprogramming after lead exposure (Table 4).

**Table 4:** Proteins identified in the SCV 1 strain whose relative quantity has undergone a statistically significant change in their abundance after lead exposure

Protein	UniProtKB	Gene	Fold Change**
DNA-directed RNA polymerase subunit omega	Q7NRL2	rpoZ	− 2.6
DNA mismatch repair	Q7NRW7	mutS	− 2.4
ABC superfamily (Glutamate/aspartate transporter)	Q7P1U5	gltL	− 1.8
RidA family	Q7NSM8	CV_3393*	− 1.8
50 S ribosomal protein L3 glutamine methyltransferase	Q7NW19	prmB	− 1.6
RNA polymerase sigma factor	Q7NRL8	rpoD	− 1.4
UDP-3-O-acyl-N-acetylglucosamine deacetylase	Q7NQ05	lpxC	− 1.4
Octaprenyl-diphosphate synthase	Q7NZS4	CV_0768	− 1.3
Leucine–tRNA ligase	Q7P0R1	leuS	− 1.2
Probable sun-like	Q7NQ78	CV_4262	− 1.2
Ribonucleoside-diphosphate reductase	Q7NVQ5	nrdA	− 1.2
4-hydroxy-3-methylbut-2-en-1-yl diphosphate synthase (flavodoxin)	Q7NS88	ispG	− 1.1
Acetyltransferase component of pyruvate dehydrogenase complex	Q7P0N9	aceF	1.1
Protein RecA	Q7NXL9	recA	1.1
30 S ribosomal protein S11	Q7NQH5	rpsK	1.2
ATP synthase gamma chain	Q7P096	atpG	1.2
Chemotaxis	Q7NSI0	cheV2	1.2
Dihydrolipoyllysine-residue succinyltransferase component of 2-oxoglutarate dehydrogenase complex	Q7NZ50	sucB	1.2
GLSA_CHRVO Glutaminase	Q7NQH9	rpoZ	1.2
Single-stranded DNA-binding	Q7NWU0	ssb	1.2
Uracil-DNA glycosylase	Q7P1I1	ung	1.2
Molybdate-binding periplasmic	Q7NRQ4	modA	1.3
3-deoxy-manno-octulosonate cytidylyltransferase	Q7NSS6	kdsB	1.4
Chaperone protein HscA homolog	Q7NZ33	hscA	1.4
Lipoprotein	Q7NVP5	CV_2297	1.4
Peptidylprolyl isomerase	Q7NTX1	CV_2932	1.4
Phosphomannomutase	Q7NW18	algC	1.4
Probable binding protein component of ABC transporter	Q7NZ25	CV_1097	1.4
Tryptophan synthase alpha chain	Q7NUD9	trpA	1.4
Glycerol kinase	Q7P1G2	glpK	1.5
Nucleoid-associated	Q7NXL5	CV_1611	1.5
Probable outer membrane multidrug resistance lipoprotein	Q7P001	CV_0768	1.5
Chromosome partitioning	Q7P0U1	soj	1.6
HIT domain-containing	Q7NQ97	CV_4243	1.6
Maltose O-acetyltransferase	Q7NVT7	maa	1.8
Translation initiation factor IF-1 1	Q7NWT1	infA1	1.9
Probable amidase from nicotinamidase family	Q7NQA1	CV_4239	2.1
RNA polymerase-binding transcription factor	Q7P1N9	dksA	2.2
Probable amino acid ABC transporter, periplasmic amino acid-binding	Q7P1U2	CV_0120	2.4
Iron-sulfur cluster assembly scaffold protein IscU	Q7NZ29	nifU	2.7
dITP/XTP pyrophosphatase	Q7NZJ6	CV_0926	2.8
Dihydroorotase	Q7NQ12	pyrC	2.9
Cysteine synthase	Q7NTM9	cysM	16

* Reannotated protein after functional alignment. ** The data generated from the analysis in the Perseus program using the log2 of the LFQ intensity of at least 2 replicates of the test and two replicates of the negative control, applying the Student’s t-test with *p* < 0.05. The data consists of converting the log2 of the fold change of the respective strains to absolute values which indicate the number of times that a given protein was regulated after lead exposure, with positive values referring to up-regulation and negative values referring to down-regulation

### Protein-protein interaction network analysis

Next, analyzes of the protein-protein interaction network were carried out to systematize, analyze and integrate the information generated through the *C. violaceum* proteome, especially regarding its bioremediation capacity against lead. The data presented represents a protein-protein interaction network consisting of 187 nodes and 459 edges, which represents the metabolic relationships between each protein in the network. Furthermore, the data reveals the presence of proteins called hub-bottlenecks (bottlenecks), which are proteins (nodes) with a high number of connections with other proteins in the network, being considered essential for the flow of generated information (Fig. 3).

Figure 3. Protein-protein interaction network represented 187 nodes and 459 edges. The square nodes represent the hub-bottleneck proteins. The data generated regarding protein abundance and regulation were obtained from the Perseus program using the log2 of the LFQ intensity of at least 2 replicates of the test and two replicates of the negative control, applying the Student’s t-test with *p*<0.05.

The centrality indices enabled identifying nodes which are considered essential to control the flow of genetic information between biological processes, (bottleneck proteins). based on the values referring to the number of edges associated with a node (degree) the data highlight the identification of six bottlenecks described in Table 5.

**Table 5:** Bottleneck Proteins information describing the node degree and the betweeneess index of the bottleneck

Protein	Gene ID	Node Degree	Betweenness
30S ribosomal S11	CV_4162	17	1.84
RNA polymerase sigma factor	CV_3762	17	2.22
Leucyl-tRNA synthetase	CV_0505	21	1.29
Bifunctional acetaldehyde-CoA/alcohol dehydrogenase	CV_1137	21	1.82
DNA recombination/repair protein	CV_1607	25	2.56
Phosphoenolpyruvate synthase	CV_3709	29	3.87

The greater the number of edges, the greater the number of connections of the respective nodes and the greater its degree (degree), while the betweenness values represent the number of shortest paths (minimum path) between the vertices and all others which pass through a single node. Thus, it is possible to understand which node has greater centrality, meaning it has greater importance in structuring the network.

## Discussion

Because *C. violaceum* SCV1 is reported here for the first time, direct comparisons with previous studies are not possible. Therefore, the differences observed between SCV1 and the type strain ATCC 12,472 constitute the first evidence of the adaptive characteristics of this environmental isolate under Pb exposure.

The ability for lead bioremediation presented by both *C. violaceum* strains (ATCC 12472 and SCV1) found in this study demonstrated the involved resistance mechanisms and bacterial adaptation against lead, which could be intensified under constant bacterial exposure conditions to this element (Baraúna et al. 2015; Castro et al. 2015; Alencar et al. 2019). Ecological imbalance in eutrophic environments due to the excess of elements such as carbon, nitrogen and phosphorus tend to alter the nutritional supply of organisms that live there and thereby influencing biological growth and resistance patterns to different contaminants and pollutants released into the environment, in addition to abundance, composition, virulence and microorganism survival in these ecosystems (Smith and Schindler 2009). These facts may explain why *C. violaceum* SCV1 strain showed greater acclimatization efficiency and in vitro lead removal when compared to *C. violaceum* ATCC 12,472 strain, suggesting a possible influence of the eutrophication process since the isolation of *C. violaceum* SCV1 was carried out in an environment impacted by the problem under study.

In this context, microorganisms develop mechanisms that help them to tolerate impacts such as eutrophication, or the action of metals such as lead to survive in the face of selective pressures imposed by the environment. In this sense, bio-sorption and ionic complexation stand out among these resistance mechanisms and bacterial adaptations (Rathnayake et al. 2009; Abdelatey et al. 2011; Baraúna et al. 2015).

The use of different incubation times should be interpreted according to the biological question addressed in each experiment. While proteomic and ultrastructural analyses were designed to investigate the early adaptive response of metabolically active cells to Pb exposure, lead removal was evaluated after prolonged incubation to estimate the maximum bioremediation efficiency under the experimental conditions employed. The scanning electron microscopy analysis showed that *C. violaceum* ATCC 12,472 and SCV 1 strains were able to biosorb lead in their structure, however without undergoing significant structural changes and without any differences between both strains. Lead removal was not quantified after 12 h, which precluded a direct temporal correlation between protein abundance and Pb removal efficiency. Future studies including multiple sampling times will allow a more comprehensive understanding of the temporal relationship between molecular adaptation and bioremediation performance. Furthermore, proteomic analyses revealed several proteins related to the bio-sorption and bio-molecule complexation.

### Bio-sorption and bio-molecule complexation

The proteins encoded by the following ORFs stood out when evaluating the bio-sorption and metal complexation by the respective *C. violaceum* strains: for the iron ion (FbpA [CV_1908], Cytochrome c [CV_1855], iron uptake ABC transporter [CV_3056], LeuC [CV_2784], NifU [CV_1093] identified in ATCC 12472 and Fdx2 [CV_1088], RimO [CV_1373], Edd [CV_0144], HemW [CV_0927], NuoE [CV_0945], LeuC [CV_2784], ErpA [CV_3694] described in *C. violaceum* SCV 1); for the zinc ion (Guanine deaminase [CV_0578], gmhA [CV_0357], adhE [CV_1137], GlobB [CV_1254], DdpX [CV_0902], HutI [CV_0321] present in *C. violaceum* ATCC 12472 and Guanine deaminase [CV_0578], ZnuA [CV_3064] in *C. violaceum* SCV 1); for the magnesium (Rnc [CV_2066] in the ATCC 12472 strain and RibB [CV_4216] in *C. violaceum* SCV 1); for the manganese ion (HutG [CV_0322] identified in both strains); and for the copper ion (CutC [CV_1414] present in *C. violaceum* ATCC 12472).

Such proteins mentioned above can be considered candidates for the lead bioremediation process, since the bio-sorption and ionic complexation enable metal retention close to the cell surface and consequently reduce its concentration in the medium (Naz et al., 2015, Huang et al. 2016; Vijayaraghavan and Yun 2008; Babu et al. 2015; Chen et al. 2016).

### Efflux, uptake and ionic transport

Another important adaptation and bacterial resistance mechanism to stressors are efflux, uptake and ionic transport. In addition to being essential to bacterial survival, these mechanisms play a decisive role in bioremediation processes. Proteins related to TonB were identified in both *C. violaceum* strains after lead exposure, as described in *C. violaceum* ATCC 12,472 (CV_1982, CV_1971, CV_0400) and in *C. violaceum* SCV 1 (CV_3896, CV_0077) strains, which can play functions associated with the capture and active bio-molecule transport associated with the response to oxidative stress (Hickman et al. 2017; Sarver et al. 2018).

Proteins related to the efflux and ionic uptake process may also play an important role in the metal bioaccumulation and the consequent bioremediation of these elements (Akhter et al. 2017). In this study, proteins relevant to bioaccumulation and intracellular bio-molecules uptake were identified post lead exposure. Such proteins were more present in *C. violaceum* SCV 1 strain, such as Probable iron uptake ABC transporter periplasmic solute-binding protein (CV_1855), Efflux pump membrane transporter (CV_0434), Alkylphosphonate uptake (CV_0443) and Probable outer membrane multidrug resistance lipoprotein (CV_0710), being considered possible candidates for lead bioremediation.

In addition, proteomic analyzes revealed the importance of transport systems in the lead bioremediation process which are mediated by the same mechanisms used to transport metabolically important ions such as potassium, magnesium and sodium (Perpetuo et al. 2011). Transport mechanisms, especially related to the ABC system were found in the present study when evaluating the molecular profile and the differential identification of *C. violaceum* proteins. Proteins considered relevant products for lead bioremediation were identified in both *C. violaceum* strains, such as CV_2520, CV_1195, CV_1681, CV_3161 and CV_1855 identified in ATCC 12,472; and CV_1097, CV_0120, CV_3936, CV_3943 in SVC 1, all of them are putative components of ABC transporter mechanism.

Furthermore, 11.7% of the proteins in *C. violaceum* ATCC 12,472 presented functions correlated to the bio-molecules transport, whereas the number of proteins with transport functions in *C. violaceum* SCV 1 was 22.3%. This fact may explain the better acclimatization of *C. violaceum* SCV 1 against lead and consequent bioremediation capacity compared to *C. violaceum* ATCC 12,472. According to Grangeiro et al. (2004), there is a direct relationship between the transport systems present in *C. violaceum* and their adaptive capacity to different environmental conditions. Also, according to these authors, the bacteria would undergo reprogramming in metabolism in stress situations, and there would be a greater need for the synthesis of proteins which transport biomolecules. Therefore, it can be assumed that the significant number of carrier proteins, especially in *C. violaceum* SCV 1, is due to lead-induced metabolic reprogramming.

### Sensory and regulatory systems

Therefore, organisms generally tend to alter their metabolism in a stressful situation, diverting resources destined for cell growth and proliferation to mechanisms which ensure cell survival (Schimel et al. 2007). Thus, it is essential to activate sensory and regulatory systems in stressful conditions. The sensory protein is responsible for identifying the presence of the stimulus from the environment or the molecules of the stressor, while the regulatory protein is responsible for mediating the activation of the cellular response in a process called signal transduction (Shpakov and Pertseva 2008; Podgornaia and Laub 2013).

The presence of many ORFs that act in signal transduction mechanisms and cellular communication has been reported in *C. violaceum* (Vasconcelos et al., 2003). It is also noteworthy that modulation of the cellular response in most cases occurs by transcriptional regulation through activating transcription factors which are proteins capable of binding to DNA and modulating the transcription efficiency of target genes (Salama and Stekel 2010; Podgornaia and Laub 2013). Several transcriptional regulators associated with a protection mechanism against reactive oxygen species were identified in both *C. violaceum* strains post lead treatment in this study, among which we can mention: LuxR (CV_2323), Crp/Fnr (CV_2987), AraC-type DNA-binding (CV_3088), Kdp operon (CV_1595), GntR (CV_2956), MerR (CV_1779) identified in *C. violaceum* ATCC 12,472; or the RNA polymerase-binding (CV_0174), nitrogen assimilation regulatory response (CV_3592), LysR (CV_3626), AraC (CV_4141), and MarR (CV_3905) present in *C. violaceum* SCV 1. Such transcriptional regulators act in regulating enzymes such as superoxide dismutase, porins, peroxidase and DNA-binding protein, and can favor lead bioremediation through signaling and cellular communication mechanisms.

### Repair mechanisms in C. violaceum

In addition to regulatory systems, repair mechanisms are essential for bacterial survival in stressful conditions and reinforce the possibility of using the respective organism for bioremediation. Several proteins essential to the repair of genetic material were identified in this study, namely, the DNA repair RecO protein (CV_2069) was exclusively identified in *C. violaceum* ATCC 12,472, whereas the oxidative inactivated repair protein - Peptide methionine sulfoxide reductase (CV_2325) was exclusively identified in *C. violaceum* SCV 1. Several proteins which are common to both strains were also identified, such as DNA repair RadA (CV_2974), Transcription-repair-coupling factor (CV_1146), DNA repair RecN (CV_2321), Recombination RecR (CV_1612), and DNA recombination/repair RecA (CV_1607), which act directly on the cellular genetic material, giving it better integrity. In addition, the presence of the single-stranded DNA-binding protein - SSB (CV_1889) was observed in both *C. violaceum* strains, which is essential during the DNA replication, repair and recombination processes, and which together with RadA, RecN, RecR and RecA proteins is part of the homologous recombination mechanisms. The SSB protein mainly acts in nucleotide excision and DNA strand re-synthesis (Duarte et al., 2004).

Mismatch DNA repair proteins were also described in *C. violaceum* ATCC 12472 and SCV 1 strains, for example mismatch repair mutS (CV_3661) and DNA mismatch repair endonuclease MutL (CV_1342). The mismatch repair proteins associated with DNA replication generally have a better understanding in prokaryotic organisms and involve complexes protein, such as MutL, MutS, MutH (Li 2008). Several proteins related to biomethylation were also found in this study, constituting a fact which reinforces the bioremediation potential of both *C. violaceum* strains in the presence of lead. Toxic metallic compounds can be transformed into less toxic forms through biomethylation. For example, lead can be methylated via biomethylation under aerobic and/or anaerobic conditions. The methyl-transferase (CV_0170), tRNA (guanine methyl-transferase (CV_3673), Ribosomal RNA small subunit methyl-transferase J (rsmJ) and Ribosomal RNA large subunit methyl-transferase E (CV_3798) proteins were identified in *C. violaceum* ATCC 12472 in this study, whereas Ribosomal protein S12 methyl-thiotransferase RimO (CV_1373) and Hydroxymethylpyrimidine kinase (CV_0151) were found in the *C. violaceum* SV1.

Some significant proteins encoded only in *C. violaceum* SCV 1 were also found, such as the methionine sulfoxide reductase protein peptide (CV_2325), which mainly acts in repairing proteins inactivated by the oxidation process. Proteins related to chemotaxis processes, flagellar motility and ion efflux systems were also found such as BON domain-containing proteins (CV_3758), Flagellar brake (CV_0609) and Probable outer membrane multidrug resistance lipoprotein (CV_0710). This fact may hypothetically explain a better response of *C. violaceum* SCV 1 against higher lead concentrations, as described in the respective growth curves and comparative scanning electron microscopy between both strains.

### Biotechnological products for the lead bioremediation

Furthermore, a significant number of uncharacterized proteins were identified in both *C. violaceum* strains after lead treatment, for which 39.1% and 52.9% had an Absent Preserved Domain or Domain with Unknown Function in *C. violaceum* ATCC 12,472 and *C. violaceum* SCV 1, respectively. This fact suggests the possibility that such proteins may act as biotechnological products for the lead bioremediation, since possible correlations with bio-molecules transport mechanisms were found after evaluating the genetic context of these proteins with the following proteins being described - (CV_4324, CV_3560 - in *C. violaceum* ATCC 12472 and CV_2519, CV_0338, CV_0397, CV_0063 - in *C. violaceum* SCV 1), transcriptional regulation (CV_2383 - in *C. violaceum* ATCC 12472 and CV_3773, CV_0063 - in *C. violaceum* SCV 1) and biomethylation and chemotaxis (CV_0397 – in *C. violaceum* SCV 1).

In addition, systems biology analyzes were performed based on the interaction between the identified proteins, which in turn can be represented through an interaction network (Pons and Latapy 2006).

Six proteins were considered essential for controlling the information flow can be highlighted when analyzing the protein interaction networks identified in both strains. Such proteins are called bottlenecks, which establish connections with several other proteins. The bottlenecks are highly connected nodes endowed with structural importance for the network maintenance and homeostasis. These proteins are considered targets for molecular interventions, especially when associated with important metabolic pathways, since their inactivation can disrupt a large part of the network around them, interfering with metabolism (Cho et al. 2004). The bottlenecks described in this study mainly performed functions associated with repair, response to oxidative stress and acclimatization metabolism.

The DNA recombination/repair protein - RecA (CV_1607) stood out among the identified bottlenecks. This protein plays an important role in a complex damage repair system for genetic material, preserving the balance between different metabolic processes and the genome stability. Such a protein reveals the efficient adaptive *C. violaceum* capacity post lead exposure, increasing the survival capacity of this organism and giving it protection against the damage caused by lead in its genetic material (Janion 2008; Bakhlanova et al. 2016).

Another important bottleneck found in the network was the RNA polymerase sigma factor protein - RpoD (CV_3762), which is essential for initiating the bacterial transcription, signal transduction and cellular communication processes, enabling specific binding of RNA polymerase to the promoters of their respective genes, and which is essential given the acclimatization and bacterial resistance mechanisms (Ho and Ellermeier 2012; Burton and Burton 2014).

Furthermore, the ribosomal 30 S ribosomal hub-bottleneck protein S11 - RpsK (CV_4162) was also found, which is fundamental for protein synthesis, and is also responsible for connecting several different RNA helices of the 16 S rRNA. A study carried out by Takada et al. (2014) suggests that this protein plays an important role in the *Bacillus subtilis* cellular motility, so that in this study, this protein may be acting in the context of *C. violaceum* motility against the adverse conditions imposed by lead. The leucyl-tRNA synthetase - LeuS (CV_0505) was also identified, which may be acting decisively in response to the damage caused by lead in the *C. violaceum* genetic material, consequently giving it a better adaptive response and resistance as evidenced by the bioremediation potential described in this study. This protein generally reacts with three substrates (ATP, leucine and tRNALeu), resulting in the tRNALeu aminoacylation. This protein performs an error correction function in bacteria (editing domain), thus guaranteeing the specificity of the cognate amino acids during the aminoacylation process. Leucyl-tRNA synthetase performs a pre-transfer (amino acid hydrolysis - cyclic adenosine monophosphate) and post-transfer (amino acid hydrolysis - tRNA) to prevent non-cognate amino acids from being incorrectly incorporated into newly synthesized proteins, making the translation process precise and efficient (Tan et al. 2010; Jakubowski 2012).

Another important bottleneck found in the network was the phosphoenolpyruvate synthase protein - PpsA (CV_3709). In this study, this protein may be providing *C. violaceum* with an energy supply and consequent survival against lead, increasing its adaptive versatility and reinforcing its bioremediation potential. The phosphoenolpyruvate synthase protein generally plays an important role in ATP formation by substrate-level phosphorylation and is considered an efficient tool to meet the demand for ATP in organisms exposed to oxidative stress conditions (McCormick and Jakeman 2015). Thus, it is observed that the phospho-transfer networks become critical for cellular energy homeostasis in these conditions, with an increase in the demand for ATP, or ineffective phosphorylation and drastic interruption in ATP production (Hall and Ji 2013; Auger and Appanna 2015).

Although the present findings highlight the biotechnological potential of *C. violaceum* for Pb bioremediation, its practical application should be evaluated within an appropriate biosafety framework, as this species is recognized as an opportunistic pathogen under specific clinical conditions (Durán and Menck 2001). Therefore, strategies involving the environmental release of viable cells require careful risk assessment, particularly given the potential ecological and clinical implications associated with the use of opportunistic microorganisms. In this context, the molecular candidates identified in this study may provide safer alternatives for developing bioremediation technologies, including the use of isolated biomolecules, purified enzymes, immobilized biomass, non-viable cells, or engineered microbial systems expressing selected *C. violaceum* proteins (Kumar et al., 2022; Singh et al., 2022).

Thus, the characterization of Pb-responsive mechanisms in *C. violaceum* is expected to contribute not only to a better understanding of bacterial adaptation to metal stress but also to the development of environmentally sustainable and biosafe bioremediation technologies. The evidence highlighted in this study regarding metabolic reprogramming in *C. violaceum* against lead through the adaptive and resistance behavior of the respective bacteria, as well as its ability to remove lead in vitro, or even the identification of several proteins correlated with bio-molecule transport mechanisms, repair, motility, chemotaxis, signal transduction, bio-sorption, bioaccumulation and bio-methylation, point to *C. violaceum* as an alternative and promising tool for the bioremediation of environments impacted by lead and the consequent control or reversal of this environmental problem.

## Conclusion

This study aimed to assess on proteomics and proposition of *C. violaceum* in lead bioremediation on a strain that has been environmentally isolated by the first time from a Brazilian area with natural occurrence of lead. The changes in relative abundance and protein profile of both *C. violaceum* strains when exposed to the metal suggest reprogramming of its metabolism. *C. violaceum* SCV 1 showed better acclimatization capacity and a bioremediation response to lead when compared to the ATCC 12,472 strain. Several proteins which are candidates for lead bioremediation were described in this study, and were mainly associated with bio-sorption, biomethylation, bioaccumulation and transport mechanisms. The lead bio-sorption mechanism of bacteria was also observed according to scanning electron microscopy analyses, being reinforced by the description of numerous proteins related to the iron, zinc, magnesium, manganese and copper complexation. Bio-molecule transport processes mainly related to the ABC transport system and ion channel formation were also described. Six bottlenecks stood out when analyzing the interaction networks of the respective proteins identified in *C. violaceum* ATCC 12,472 and SCV 1 strains, which mainly performed functions associated with repair, stress response and acclimatization metabolism. Numerous proteins with Missing Conserved Domain or Domain with Unknown Function were also highlighted, in which the evaluated genetic context maintained a possible correlation with bio-molecule transport, transcriptional regulation, biomethylation and chemotaxis mechanisms. Such observation opens the possibility of studying and unraveling these proteins as possible candidates for lead bioremediation and other metals in further scientific studies. Thus, the data evaluated in this study show the potential of *C. violaceum* and reveal its applicability as an alternative for the remediation of environments impacted by lead. Future studies should focus on the functional characterization and validation of the Pb-responsive proteins and adaptive mechanisms identified in this study to better understand their roles in Pb tolerance and biosorption. Such investigations may support the development of environmentally effective and biosafe biotechnological strategies for the remediation of Pb-contaminated environments while minimizing the risks associated with the environmental release of viable *Chromobacterium violaceum* cells.

## Supplementary Information

Below is the link to the electronic supplementary material.

Supplementary Material 1

## Data Availability

No datasets were generated or analysed during the current study.
