# Supplementary material for: Bioprospecting Chromobacterium violaceum for bioremediation: an alternative to environmental lead pollution
Source: World J Microbiol Biotechnol. 2026 Sep 4;42(9):515. doi: 10.1007/s11274-026-05190-8 (PMC13545060; doi:10.1007/s11274-026-05190-8)
Supplement: Supplementary file 1 [file 11274_2026_5190_MOESM1_ESM.docx]

Supplementary figures

 Supplementary figure 1. Enzymatic classification of proteins identified in *C. violaceum* after lead-induced oxidative stress. Classification of the main catalytic activities described in the proteins identified in *C. violaceum* after exposure to lead. The data take into account the total amount of proteins identified after lead exposure in the ATCC 12472 (n = 104) and SCV 1 (n = 103) strains, considering that two or more catalytic activities may be present in the same protein.

Supplementary figure 2. Molecular function of proteins identified in *C. violaceum* after lead-induced oxidative stress. Main molecular functions described in the proteins identified in the ATCC 12472 and SCV 1 strains after lead exposure. The figure describes molecular characteristics associated with adsorption, binding, transport, regulation, resistance, organization and signaling performed by the respective proteins. The data take into account the total of proteins identified after lead exposure in the ATCC 12472 (n = 104) and SCV 1 (n = 103) strains, considering that two or more molecular functions may be present in the same protein.
